# Structural characterization of human *de novo* protein NCYM and its complex with a newly identified DNA aptamer using atomic force microscopy and small-angle X-ray scattering

**DOI:** 10.3389/fonc.2023.1213678

**Published:** 2023-11-23

**Authors:** Seigi Yamamoto, Fumiaki Kono, Kazuma Nakatani, Miwako Hirose, Katsunori Horii, Yoshitaka Hippo, Taro Tamada, Yusuke Suenaga, Tatsuhito Matsuo

**Affiliations:** ^1^ Laboratory of Evolutionary Oncology, Chiba Cancer Center Research Institute, Chiba, Japan; ^2^ Institute for Quantum Life Science, National Institutes for Quantum Science and Technology, Chiba, Japan; ^3^ Graduate School of Medical and Pharmaceutical Sciences, Chiba University, Chiba, Japan; ^4^ Innovative Medicine CHIBA Doctoral WISE Program, Chiba University, Chiba, Japan; ^5^ All Directional Innovation Creator Ph.D. Project, Chiba University, Chiba, Japan; ^6^ Digital Healthcare Business Development Office, NEC Solution Innovators, Ltd., Tokyo, Japan; ^7^ Laboratory of Precision Tumor Model Systems, Chiba Cancer Center Research Institute, Chiba, Japan; ^8^ Graduate School of Science, Chiba University, Chiba, Japan

**Keywords:** *de novo* protein, NCYM, DNA aptamer, solution structure, AFM, SAXS

## Abstract

NCYM, a Homininae-specific oncoprotein, is the first *de novo* gene product experimentally shown to have oncogenic functions. NCYM stabilizes MYCN and β-catenin via direct binding and inhibition of GSK3β and promotes cancer progression in various tumors. Thus, the identification of compounds that binds to NCYM and structural characterization of the complex of such compounds with NCYM are required to deepen our understanding of the molecular mechanism of NCYM function and eventually to develop anticancer drugs against NCYM. In this study, the DNA aptamer that specifically binds to NCYM and enhances interaction between NCYM and GSK3β were identified for the first time using systematic evolution of ligands by exponential enrichment (SELEX). The structural properties of the complex of the aptamer and NCYM were investigated using atomic force microscopy (AFM) in combination with truncation and mutation of DNA sequence, pointing to the regions on the aptamer required for NCYM binding. Further analysis was carried out by small-angle X-ray scattering (SAXS). Structural modeling based on SAXS data revealed that when isolated, NCYM shows high flexibility, though not as a random coil, while the DNA aptamer exists as a dimer in solution. In the complex state, models in which NCYM was bound to a region close to an edge of the aptamer reproduced the SAXS data. Therefore, using a combination of SELEX, AFM, and SAXS, the present study revealed the structural properties of NCYM in its functionally active form, thus providing useful information for the possible future design of novel anti-cancer drugs targeting NCYM.

## 1 Introduction

*NCYM*, a *cis*-antisense gene of *MYCN*, encodes a Homininae-specific oncoprotein (1, 2). In human neuroblastomas, *NCYM* is always co-amplified with *MYCN*, and its expression level is associated with poor prognosis (1). NCYM stabilizes MYCN via inhibition of GSK3β, whereas MYCN stimulates both *MYCN* and *NCYM* transcription (1). This feedback loop contributes to the maintenance of high levels of both MYCN and NCYM expressions in *MYCN*-amplified neuroblastomas (1, 2). NCYM enhances the metastasis of neuroblastomas (1) possibly via inhibition of apoptotic cell death (1, 3, 4) and/or regulation of stemness (5, 6). Furthermore, NCYM has been shown to be associated with progression of adult cancers (2, 7). Therefore, NCYM is a promising target protein for anti-cancer therapy. However, the difficulty in determining its structure hinders drug design (8).

*NCYM* is a newly evolved coding gene that originated from *MYCN* promoter region during the evolution of the Homininae (1, 2). New genes originating from non-genic regions are known as *de novo* gene birth (9–11), and NCYM is the first *de novo* gene product experimentally shown to have oncogenic functions. Owing to their *de novo* emergence, *de novo* proteins show no homology to known genes and do not have any domains or motifs. The amino acid sequence of *de novo* proteins is similar to a random sequence (12), although a recent report identified the difference between *de novo* proteins and unevolved random-sequence counterparts in that *de novo* proteins exhibit moderately higher solubility in cells (13). Four *de novo* proteins have been structurally characterized to date: Bsc4 (14), NCYM (8), Goddard (15), and AFGP8 (16); however, mainly because of their highly disordered nature, none of the complete structures have been determined. Upon binding to the ice surface, the local structure of the antifreeze glycoprotein AFGP8 make a transition from a disordered to an ordered state (16), indicating the possibility of significant ordering of *de novo* proteins via complex formation with binding partners. Consistent with an earlier prediction that NCYM binds to DNA (17), we have previously found that benzonase treatment significantly improves the solubility of NCYM (8). These observations led us to identify DNA aptamers that bind specifically to NCYM and to consider that analysis of the complex of NCYM and DNA aptamers may contribute to the characterization of the structural dynamics of NCYM.

Here, three types of DNA aptamers were identified by systematic evolution of ligands by exponential enrichment (SELEX) and their interactions with NCYM were characterized by atomic force microscopy (AFM). Moreover, the structure of the NCYM-DNA complex of a representative DNA aptamer (named “No. 1”), which enhances interaction between NCYM and GSK3β, was analyzed using small-angle X-ray scattering (SAXS).

## 2 Materials and methods

### 2.1 Aptamer selection procedure

SELEX was performed as previously reported with some modifications (18, 19). Dynabeads™ MyOne™ Carboxylic Acid (CA) magnetic beads (Invitrogen, Waltham, MA) were used for NCYM solidification to segregate NCYM-binding DNA molecules from the non-binding molecules. The target beads were prepared by an amine coupling reaction using 1-ethyl-3-(3-dimethylaminopropyl) carbodiimide hydrochloride (EDC; Thermo Fisher Scientific, Waltham, MA, USA) according to the manufacturer’s instructions and washed with the selection buffer [SB; 40 mM HEPES (pH 7.5), 125 mM NaCl, 5 mM KCl, 1 mM MgCl_2_, and 0.01% Tween 20]. Briefly, the CA magnetic beads were washed twice with 500 μl of 100 mM 2-Morpholinoethanesulfonic acid mono hydrate (MES) buffer at pH 6.0. After added 50 μl of 100 mM MES buffer and 50 μl of EDC, and the mixture was incubated for 30 minutes at room temperature. The mixture was then mixed with 200 μg of NCYM in 100 mM MES buffer and incubated over night at room temperature to react with the amino group of NCYM and the carboxylic acid of the CA magnetic beads. Next, they were washed twice with 500 μl of PBST (0.1% Tween20 in phosphate buffered saline, PBS) and 500 μl of PBST-BSA (0.1% bovine serum albumin in PBST) was added. Before using the target beads, they were washed twice with 1 ml of SB.

An initial single-stranded DNA (ssDNA) pool, 5′-GGAATGTGGTCCCTCGCAATAAATC-(N30)- GAAATGAGCCCTTTGACCCTGTAC-3′, containing 30 random nucleotides between forward (Fw) and revers (Rv) primer region was purchased from Integrated DNA Technologies (Tokyo, Japan). The selection of aptamers was performed starting from 4.5 nmol of DNAs (~10^15^ molecules) in 100 μl of SB. The pool was mixed for 15 minutes with 250 μg of target beads at 25°C. The beads were then washed with SB, and the bound ssDNA was eluted with 7 M urea. After recovery of the eluted ssDNA using Rv primer beads, polymerase chain reaction (PCR) was performed with KOD Dash DNA polymerase (Toyobo, Osaka, Japan), a forward (Fw) primer (5’-GGAATGTGGTCCCTCGCAATAAATC-3’) and a reverse (Rv) primer (5’-GTACAGGGTCAAAGGGCTCATTTC-3’) with modification by the 5’-biotin. Next, the amplified double stranded DNA (dsDNA) was bound to MyOne SA C1 magnetic beads, and the Fw chain (ssDNA) was eluted with 0.02M NaOH. The ssDNA was used for the next round.

After eight rounds of selection, the frequency of ssDNA sequences was determined by next-generation sequencing (NGS) from rounds 3 to 8 of SELEX using a MiniSeq System (Illumina, San Diego, CA, USA). Sequencing data were preprocessed by using the program of PRINSEQ++ (20) and adopted above 99.9% of the base calling accuracy (Q score of 30 and above).

### 2.2 Bio-layer interferometry

All the Bio-Layer Interferometry (BLI) measurements were performed at 25°C using an Octet® RED96e system (Sartorius AG, Goettingen, Germany). All the samples were placed in a 96 well microplate and the sample volume was 200 μl/well. The microplate was shaked at 1,000 rpm during the measurement. As a ligand, each aptamer with 5’-biotin modification was immobilized on an Octet® SA biosensor chip (Sartorius). For kinetics analysis, different concentrations of NCYM (25-400nM) were used. The dissociation constants between the aptamer and NCYM were calculated using a simple 1:1 biomolecular interaction model according to the manufacturer’s instructions.

### 2.3 Construction of DNA frame structures and introduction of aptamers for AFM imaging

To gain insights into the mechanism of interactions between NCYM and the aptamers obtained by SELEX, NCYM-aptamer binding was studied at the single molecule level using AFM. Scaffolds prepared using the DNA origami method were used for this purpose. The DNA origami method allows the creation of structures of any shape and the introduction of functional molecules anywhere in the structure. Therefore, single-molecule observation using DNA origami structures is suitable for evaluating biomolecules and has been used to observe various molecules (21–29). In this study, we used a DNA frame structure (21). The DNA frame contains a space inside, and dsDNA can be introduced into any sequence.

DNA frames were prepared as previously described (21). A solution containing 10 nM M13mp18 ssDNA (tilibit nanosystems GmbH, Germany), 25 nM staples (2.5 eq), 20 mM Tris-HCl (pH 7.4), 10 mM MgCl_2_, 1mM EDTA was prepared and annealed at a rate of -1°C per minute from 85°C to 15°C.

Aptamers and DNA oligos for the DNA frame were purchased from Eurofins Genomics K.K. (Tokyo, Japan) and used without further purification. In this study, three types of aptamers, which showed high affinity among the aptamers obtained by SELEX, were employed, and the sequences of which for AFM observation are as follows:

No. 1: 5’-GGAATGTGGTCCCTCGCAATAAATCTATGTACGTTATTCCCCTTTGACC
AATGCTGAAATGAGCCCTTTGACCCTGTAC **TTTT** TTTCCAGCGGGACTAGCGCGTTGCTCCTCACT-3’
No. 2: 5’-GGAATGTGGTCCCTCGCAATAAATCGGGGAGGGAGGGTGGGGGCGGT
GGGAGGTGGAAATGAGCCCTTTGACCCTGTAC**TTTT** TTTCCAGCGGGACTAGCGCGTTGCTCCTCACT-3’
No. 3: 5’-GGAATGTGGTCCCTCGCAATAAATCGGGCGTTGTGGAGGGGGCGGTG
GGTGGGGGGAAATGAGCCCTTTGACCCTGTAC**TTTT** TTTCCAGCGGGACTAGCGCGTTGCTCCTCACT-3’

All aptamers had a dsDNA complement sequence (underlined) added via a TTTT sequence (bold) at the 3’ end for introduction into the DNA frame.

The secondary structures of the aptamers were predicted using RNAfold [http://rna.tbi.univie.ac.at/cgi-bin/RNAWebSuite/RNAfold.cgi]. We used the default settings: minimum free energy (MFE) and partition function, yes; avoid isolated base pairs, yes; incorporate G-Quadruplex formation into the structure prediction algorithm, yes; dangling end options, dangling energies on both sides of a helix in any case; energy parameters, DNA parameters (Mathews model, 2004) at 25 or 38°C with a salt concentration of 1.021M.

The resultant dsDNAs were incorporated into the frame structures described above and observed in the presence of NCYM (**Figure S1**), which was purified according to a previous method (1).

### 2.4 Truncation and mutation of the DNA aptamer No. 1

To identify where the aptamers interact with NCYM, we prepared three analogs (short 1, short 2, and short 3) of the No. 1 aptamer based on the secondary structure prediction by RNAfold. The sequences used are as follows:

short 1: 5’-TGGTCCCTCGCAATAAATCTATGTACGTTATTCCCCTTTGACCAATGC
TGAAATGAGC**TTTT** TTTCCAGCGGGACTAGCGCGTTGCTCCTCACT-3’
short 2: 5’-GGAATGTGGTCCCTCGCAATAAATCTATGTACGTTATTCCCCTTTGAC
CAATGCCCTTTGACCCTGTAC**TTTT** TTTCCAGCGGGACTAGCGCGTTGCTCCTCACT-3’
short 3: 5’-GGAATGTGGTCCCTCGCATCCCCTTTGACCAATGCTGAAATGAGCCCT
TTGACCCTGTAC**TTTT** TTTCCAGCGGGACTAGCGCGTTGCTCCTCACT-3’

short 1, short 2, and short 3 lack the 5’ end and 3’ end side (named “5’-3’-end” herein), the 3’ end stem loop, and the central stem loop of the No. 1 aptamer, respectively.

In addition, mutations were introduced into aptamer No.1 without changing its secondary structure. The following sequences are mutants of aptamer No.1, and italic font indicates the introduced mutations.

No.1 mut 1 5’-GGAATGTGGTCCCTCGC *CGCGC* ATCTATGTACG *GCGCG* CCCCTTTGACCAATGCTGAAATGAGCCCTTTGACCCTGTACTTTTTTTCCAGCGGGACTAGCGCGTTGCTCCTCACT-3’
No.1 mut 2 5’-GGAATGTGGTCCCTCGCAATAAATCTATGTACGTTATTCCCC *CCG* GACCAATGCTGAAATGAGCCCTTTGACCCTGTACTTTTTTTCCAGCGGGACTAGCGCGTTGCTCCTCACT-3’
No.1 mut 3 5’-GGAATGTGGTCCCTCGCAATAAATCTATGTACGTTATTCCCCTTTGACCAATGCTGAAATGAGCCC *GCG* GACCCTGTACTTTTTTTCCAGCGGGACTAGCGCGTTGCTCCTCACT-3’
No.1 mut 123 5’-GGAATGTGGTCCCTCGC *CGCGC* ATCTATGTACG *GCGCG* CCCC *CCG* GACCAATGCTGAAATGAGCCC *GCG* GACCCTGTACTTTTTTTCCAGCGGGACTAGCGCGTTGCTCCTCACT-3’

### 2.5 AFM imaging

The AFM images were acquired using an AFM system (NanoWizard UltraSpeed, JPK) equipped with a silicon nitride cantilever (Olympus, BL-AC40TS). In all the measurements, NCYM (100 nM) 10eq was added to the adjusted frame structures having aptamer (10 nM) and incubated at 25°C for 2h. Samples were then double-diluted in annealing buffer (20 mM Tris-HCl (pH 7.4), 10 mM MgCl_2_, 1mM EDTA), adsorbed onto fresh mica plates for 10 minutes at room temperature, and washed three times with the annealing buffer. The observations were performed using the same buffer.

### 2.6 Immunoprecipitation

20 μl of Dynabeads™ protein G (Thermo Fisher Scientific) slurry was transfered to a clean tube. The tube was placed in a magnetic separation rack for 10-15 seconds, then, the buffer was carefully removed. 2 µg of anti-GSK3β (BD Transduction Laboratories) or mouse IgG (Cell Signaling Technology, Danvers, MA) was dissolved in 200 μl of PBS (0.02% Tween 20) and the solution was added to the beads. The slurry was incubated with rotation at room temperature for 10 minutes. The beads were pelleted using a magnetic separation rack and washed three times with 200 μl of PBS, then resuspended in 100 μl of PBS. 0.17µg (3.7 pmol) of purified GSK3β (Signal Chem, Richmond, Canada), 0.044g (3.7 pmol) of NCYM (1) and DNA aptamers (1eq (3.7 pmol) or 5 eq (18.5 pmol)) were dissolved in 500μl of ice-cold PBS and incubated with rotation at 4°C for 2h. A 100 μl suspension of antibody-conjugated beads was added and incubated for 2h. The beads were pelleted using a magnetic separation rack and washed five times with 1 ml of PBS. The pellet was resuspended in 20 μl of 1X sample buffer and heated at 95°C for 5min.The beads were pelleted using a magnetic separation rack and the supernatant was subjected to the Abby analysis.

### 2.7 Abby analysis

The NCYM and GSK3β protein levels were measured using a capillary electrophoretic-based immunoassay (the Abby instrument; ProteinSimple, San Jose, CA, USA), according to the manufacturer’s protocol. Briefly, the samples were combined with 0.1× sample diluent buffer and 5× fluorescent master mix denaturing buffer to acquire 0.8 µg/μl loading concentration. Subsequently, the samples were denatured for 5 min at 95 °C. The primary antibodies used in this study were anti-NCYM (1) and anti-GSK3β (#9315, Cell Signaling Technology, Danvers, MA, USA). The Abby measurements were performed using a 12–230 kDa separation module with 25-min separation at 375 V, 10-min blocking, 30-min primary antibody incubation, and 30-min secondary antibody incubation (DM-001, ProteinSimple, San Jose, CA, USA). At the end of the run, the chemiluminescent signal was displayed as a virtual blot-like image and an electropherogram based on the molecular weight using Compass (ProteinSimple, San Jose, CA, USA).

### 2.8 Sample preparation for SAXS measurements

The expression and purification procedures for NCYM were modified from those described previously (8). Recombinant NCYM with glutathione S-transferase (GST) at the N-terminus was expressed in *Escherichia coli* BL21 (DE3) cells using the pGEX-6p-1 vector. The cells were cultured in Luria broth medium containing 0.1 mg/ml ampicillin at 30°C. When OD_600_ reached 0.6, protein expression was induced by 0.1 mM of isopropyl-β-D-thiogalactopyranoside, and culture was continued for 6 h at 30°C. The cells were harvested by centrifugation (3,890×*g*, 15 min, 4°C), and then stored at −30°C until purification.

The frozen cell pellets were thawed and resuspended in phosphate-buffered saline (PBS) supplemented with cOmplete EDTA-free Protease Inhibitor Cocktail (11873580001, Roche, Basel, Switzerland) and lysed by repeated sonication in ice water. The cell lysate was centrifuged at 20,000×*g*, 4°C for 20 min, then the supernatant was loaded onto GSTrap FF column (17513102, Cytiva, Marlborough, MA, USA) equilibrated with PBS, using a peristaltic pump. After washing out the unbound materials with PBS, GST-tagged NCYM (GST-NCYM) was eluted with an elution buffer (50 mM Tris-HCl and 10 mM reduced glutathione, pH 8.0). The flow-through was reloaded onto the re-equilibrated column, and the eluate was collected once more to increase the final protein yield.

The eluted GST-NCYM solution was dialyzed against a buffer containing 50 mM Tris-HCl, 100 mM NaCl, and 1 mM EDTA at pH 8.0. After adding dithiothreitol to the protein solution at a final concentration of 1 mM, the GST-tag was cleaved with 50 U/L-culture of PreScission Protease (27084301, Cytiva) for over 18 h at 4°C with gentle stirring. To exhaustively degrade the remaining nucleic acids, 6,000 U/L-culture of benzonase (71205-3CN/70746-3CN, Merck, Darmstadt, Germany) was added to the cleaved sample along with MgCl_2_ at a final concentration of 2 mM (Mg^2+^ is required for the activation of benzonase), after which the sample was dialyzed against IEX buffer (20 mM MOPS (pH 7.0) and 1 mM DTT) with 2 mM MgCl_2_.

The dialyzed sample was loaded onto a HiTrap SP HP column (17115201, Cytiva) equilibrated with IEX buffer, and eluted using a linear gradient of NaCl (0–650 mM). The fractions containing high-purity NCYM, as confirmed by SDS-PAGE, were collected and used for SAXS measurements. The GST-tag did not bind to the column and was detected in the flow-through. The final NCYM yield was 1.25 mg/L-culture, which was estimated with the molecular absorption coefficient of 280 nm ϵ_280_^0.1%^ = 0.558.

The purified NCYM was dialyzed against a buffer containing 10 mM Tris-HCl (pH8.0), 50 mM NaCl, and 5 mM DTT. Powdered DNA aptamers (No. 1) were dissolved directly in the same buffer. Samples of the NCYM-DNA complex were prepared by mixing these two kinds of solutions at the appropriate molar ratios (see below). These solutions were used for the following SAXS measurements.

### 2.9 SAXS experiment

SAXS measurements were carried out at BL40B2 in SPring-8 (Hyogo, Japan) on solution samples of NCYM (1.4 and 2.8 mg/ml), the DNA aptamer (1.6 and 5.0 mg/ml), and the NCYM-DNA complex. For the measurements of the complex, two kinds of samples, where the molar ratio of NCYM and DNA was 1:1.2 or 1:1.5 (**Table 1**), were used to extract the scattering curve of the complex by changing the relative contribution of the unbound DNA aptamers. The wavelength (λ) of the incident X-ray was 1.0 Å and the temperature was 293 K with the sample-to-detector distance of 2.2 m. A pixel detector (PILATUS3S 2 M, Dectris) was used to record the scattering patterns.

**Table 1:** Information on the measured samples of the NCYM-DNA complex.

Molar ratio (NCYM : DNA) in the sample	NCYM concentration in the sample [mg/ml]	DNA concentration in the sample [mg/ml]
1:1.2	1.3	3.2
	0.6	1.5
1:1.5	0.6	1.8

Data reduction was conducted using the software SAngler (30): The recorded two-dimensional SAXS patterns were circularly averaged to obtain one-dimensional scattering curves, corrected by the incident flux measured with an ion chamber placed upstream of the samples. The net scattering curves of the scattering particles were obtained by subtracting the scattering curves of the buffer from those of the samples with an appropriate scaling factor based on the scattering particle concentration and the partial specific volumes of proteins (0.73 cm^3^/g) or of DNA (0.53 cm^3^/g) (31). Finally, the scattering curves were normalized to the absolute scale using H_2_O as the standard (32) to estimate the molecular weight of the scattering particles.

Guinier analysis was employed to evaluate the radius of gyration (R_g_) of the scattering particle from its scattering curve. A scattering curve I(Q), where Q (=4πsinθ/λ, where 2θ is the scattering angle) denotes the momentum transfer, is represented as follows in a good approximation in the so-called Guinier region (Q･R_g_ < 1.3) (33):


(1), $$\text{I}\left(\text{Q}\right)=\text{I}\left(0\right)\text{exp}\left(-\frac{1}{3}{\text{R}}_{\text{g}}^{2}{\text{Q}}^{2}\right)$$

where I(0) [cm^-1^] denotes the forward scattering intensity, from which the molecular weight (MW) of the scattering particle is estimated from the following equation in kDa (32):


(2), $$\text{MW}=1500\times \text{I}\left(0\right)\frac{1}{\text{c}}$$

where c is the weight concentration [g/l].

For rod-like particles such as the DNA aptamer and the NCYM-DNA complex, cross-sectional Guinier analysis was applied to evaluate their cross-sectional radii of gyration (R_c_). In this case, I(Q) is approximated as:


(3), $$\text{Q}･\text{I}\left(\text{Q}\right)={\text{I}}_{\text{c}}\left(0\right)\text{exp}\left(-\frac{1}{2}{\text{R}}_{\text{c}}^{2}{\text{Q}}^{2}\right)$$

where I_c_(0) denotes the forward scattering intensity of the cross-section of the scattering particle. Application of Eq. 2 with I_c_(0) instead of I(0) yields the scattering mass per unit length.

The scattering curves taken at the lower particle concentrations were merged with those at the higher concentrations at Q = 0.1 Å^−1^ and the resultant curves were used for structural modeling. IGOR Pro software (WaveMetrics, Lake Oswego, OR, USA) was used for (cross-sectional) Guinier analyses and for processing the scattering curves.

### 2.10 Structural modeling using the *ab initio* method

Human NCYM comprises109 residues. Because NCYM was expressed with a GST-tag at its N-terminus in this study, eight residues were added to the 109 residues even after cutting the tag. For structural modeling, the program GASBOR (34) was employed, where each residue is represented by a sphere with a constant electron density. The obtained model thus consists of 117 spheres. In this study, the GASBOR runs were repeated 10 times (i.e., 10 best-fit models were obtained). For the modeling of the DNA aptamer, DAMMIF (35) was used, where a molecule is represented by an ensemble of spheres called dummy atoms. The input files required for DAMMIF were generated using AUTORG and DATGNOM (36). The maximum Q value (Q_max_) used for structural modeling was automatically determined using DATGNOM (Q_max_R_g_ < 7–8). The DAMMIF runs were repeated 10 times and the resultant 10 models were averaged, followed by filtration using DAMAVER (37). Structural modeling of the NCYM-DNA complex was carried out using the program MONSA (38), where each of the two phases is represented by a dummy atom model while each phase is assigned a designated electron density value. As an input file of MONSA, the following information is required: The values of the electron density of NCYM and the DNA aptamer were set to be 0.09 e/Å^3^ and 0.21 e/Å^3^, respectively, which are typical of these types of molecules (39). The volume fractions of NCYM and the DNA aptamer were obtained from the volumes obtained using GASBOR and DAMMIF, respectively. Using these parameters, the MONSA runs were repeated 10 times. In all of the programs above, the best-fit model, the scattering curve of which reproduces the experimental curve well, is determined by simulated annealing.

### 2.11 Scattering curve of the NCYM-DNA complex

The scattering curves of the NCYM-DNA complex recorded at 1:1.2 and 1:1.5 molar ratios were found to be superimposable despite the existence of different amounts of unbound DNA aptamers (**Figure S2**). However, as described in the Results section, NCYM molecules and the DNA aptamers exist as monomers and dimers, respectively, in solution so that the molar ratios of NCYM and the dimeric DNA aptamer in the above samples are 1:0.6 and 1:0.75, respectively, which result in the molar ratios of the complex and the excess amount of NCYM of 0.6:0.4 and 0.75:0.25 assuming that one NCYM monomer binds to a DNA dimer. Because the scattering intensity is proportional to the square of the product of the scattering contrast of a particle and its volume, the scattering contributions of excess NCYM molecules were calculated to be 0.8% and 0.4% for the 0.6:0.4 and 0.75:0.25 samples, respectively. In this estimation, the scattering contrasts of NCYM and DNA were set to be 0.09 e/Å^3^ and 0.21 e/Å^3^ as described above, and the volumes of a NCYM monomer and a DNA dimer were assumed to be 17000 Å^3^ and 68000 Å^3^ as obtained by GASBOR and DAMMIF. Thus, it appears that the scattering contribution of free NCYM monomers is negligible, which explains why the scattering curves obtained at NCYM : DNA = 1:1.2 and 1:1.5 are similar to each other within errors. This observation also excluded the possibility that the complex consists of one NCYM monomer and one monomeric DNA aptamer. Based on the above inspection, the curves obtained from the above samples reflect those of the NCYM-DNA complex. The scattering curves taken at NCYM : DNA = 1:1.2 and 1:1.5 were, therefore, averaged to improve the signal-to-noise ratio and the resultant curve was employed as the scattering curve of the NCYM-DNA complex.

## 3 Results

### 3.1 Aptamer selection by SELEX

**Figure 1:**
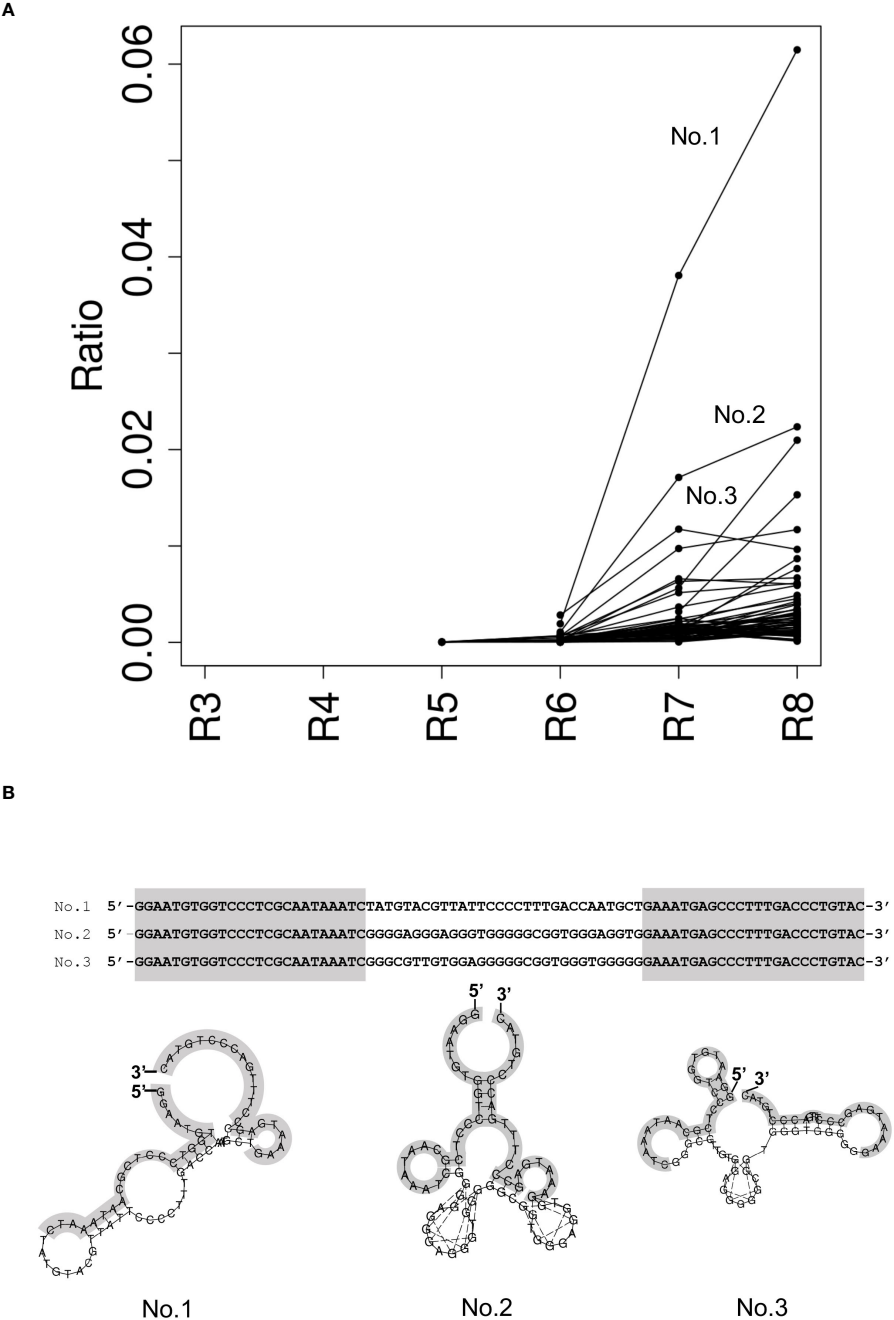
Identification of NCYM-bound aptamers. **(A)** Ratio of the enriched ssDNA sequence in the selection pool per round. Each dot represents the ratio of the enriched ssDNA sequences more than 0.001. The lines indicate the same ssDNA sequence between rounds. Top 3 enrichment sequences at round 8 were evaluated for the further structural analysis and named No. 1, No. 2, and No. 3. **(B)** Secondary structure prediction of NCYM-bound aptamer by RNAfold at 25°C. Primer sequences are colored in gray.

To identify DNA aptamers that specifically bind to NCYM, we performed SELEX and employed NGS technology to monitor the progress of the enrichment sequences that bind to the target in the selection pool. We performed the NGS analysis using an Illumina MiniSeq for the NCYM aptamer selection pools from rounds 3 to 8 to identify the aptamer candidates. The ratio of unique DNA sequences in the selection pool per round showed that DNA sequences were enriched in round 6 (**Figure 1A**). No. 1 aptamer candidate, which was the most abundant population in round 8, was quickly enriched from round 6 compared to the other DNA sequences. Guanine contents in the selection pools were increased slightly in round 8 (**Table S1**). BLI measurements and kinetic analyses showed that top three aptamers strongly bind to NCYM protein at K_D_ value in the range of 53.9 to 299 nM (**Table S2**); however, the aptamer No. 2 and 3 were predicted to form different secondary structure at 38°C, showing low structural stability at relatively high temperature compared to aptamer No.1 (**Figure 1B** and **Figure S3**).

### 3.2 AFM observation on the NCYM-DNA aptamer complexes

#### 3.2.1 DNA aptamers obtained by SELEX (No. 1, 2, and 3)

**Figure 2:**
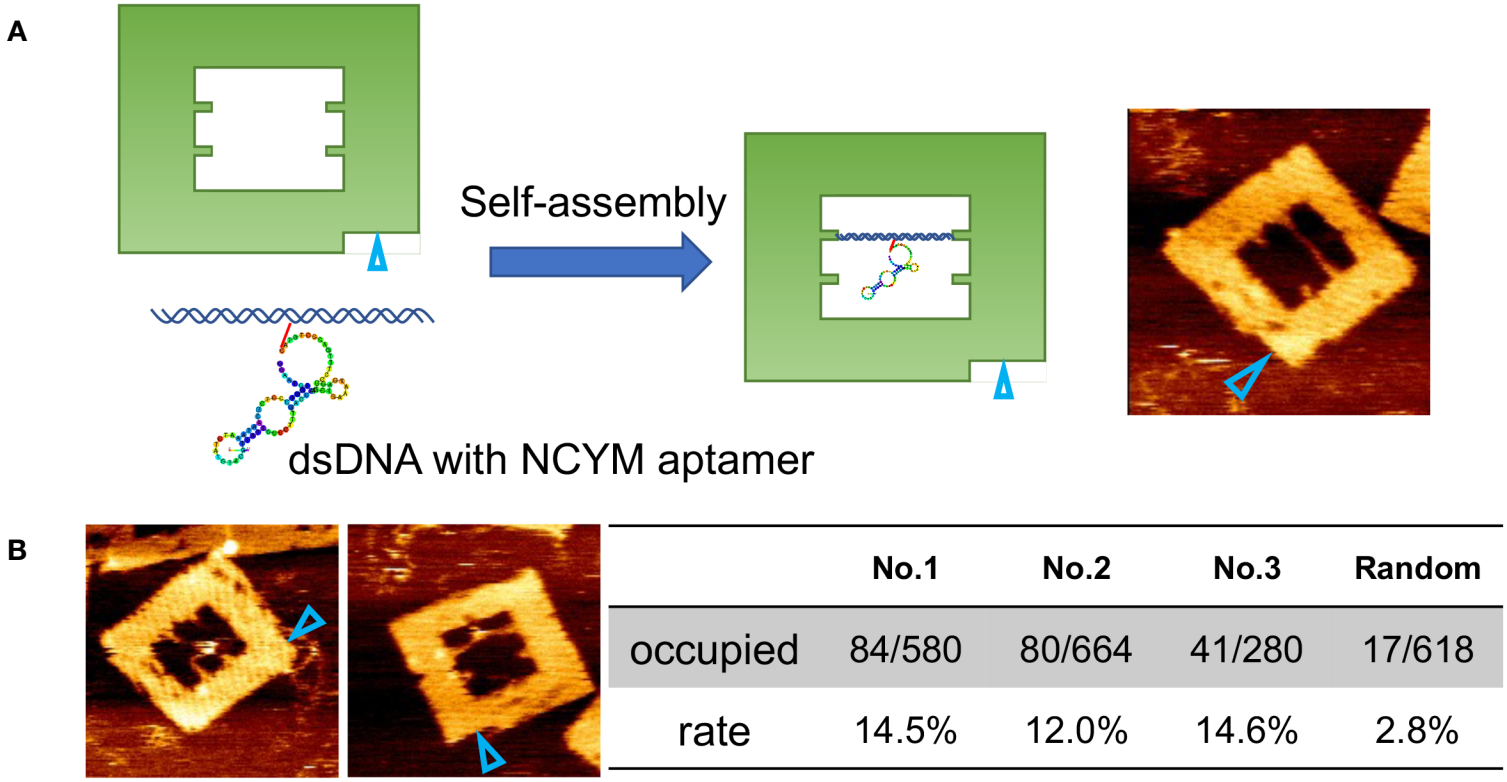
Secondary structure prediction model for aptamers and evaluation using DNA origami. **(A)** Design of DNA frame with NCYM aptamer and its AFM image. The open cyan triangles show the orientation marker. **(B)** Examples of AFM images of NCYM-aptamer No.1 complexes on DNA frames (left) and evaluation of the affinity between aptamers obtained by SELEX and NCYM (right). In the “Occupied” row, the numerator and the denominator represent the number of NCYM bound to the aptamers and the total number of aptamers, respectively. The “rate” row denotes the number fraction of NCYM bound to the aptamers calculated from the corresponding value in the “occupied” row.

To visualize the interaction between NCYM and DNA at the molecular level, we used AFM observation of the aptamer-conjugated DNA frames with or without NCYM (**Figure 2**). Because the aptamer has a large single-stranded region and is not completely fixed, only dsDNA can be seen. After adding NCYM, white dots were observed on the dsDNA (**Figure 2B**). The numbers of DNA frames with and without these white dots were counted to assess the affinity of the aptamer for NCYM (**Figure 2B**). Binding of NCYM to the No. 1 aptamer was observed in 14.5% of the DNA frames (84/580 frames). Binding was also observed for the No. 2 and No. 3 aptamers at 12.0% (80/664 frames) and 14.6% (41/280 frames), respectively. The slightly lower affinity of No. 2 compared to No.1 and No.3 is consistent with the KD value evaluated by BLI measurements.

#### 3.2.2 Truncated and mutated DNA aptamers

**Figure 3:**
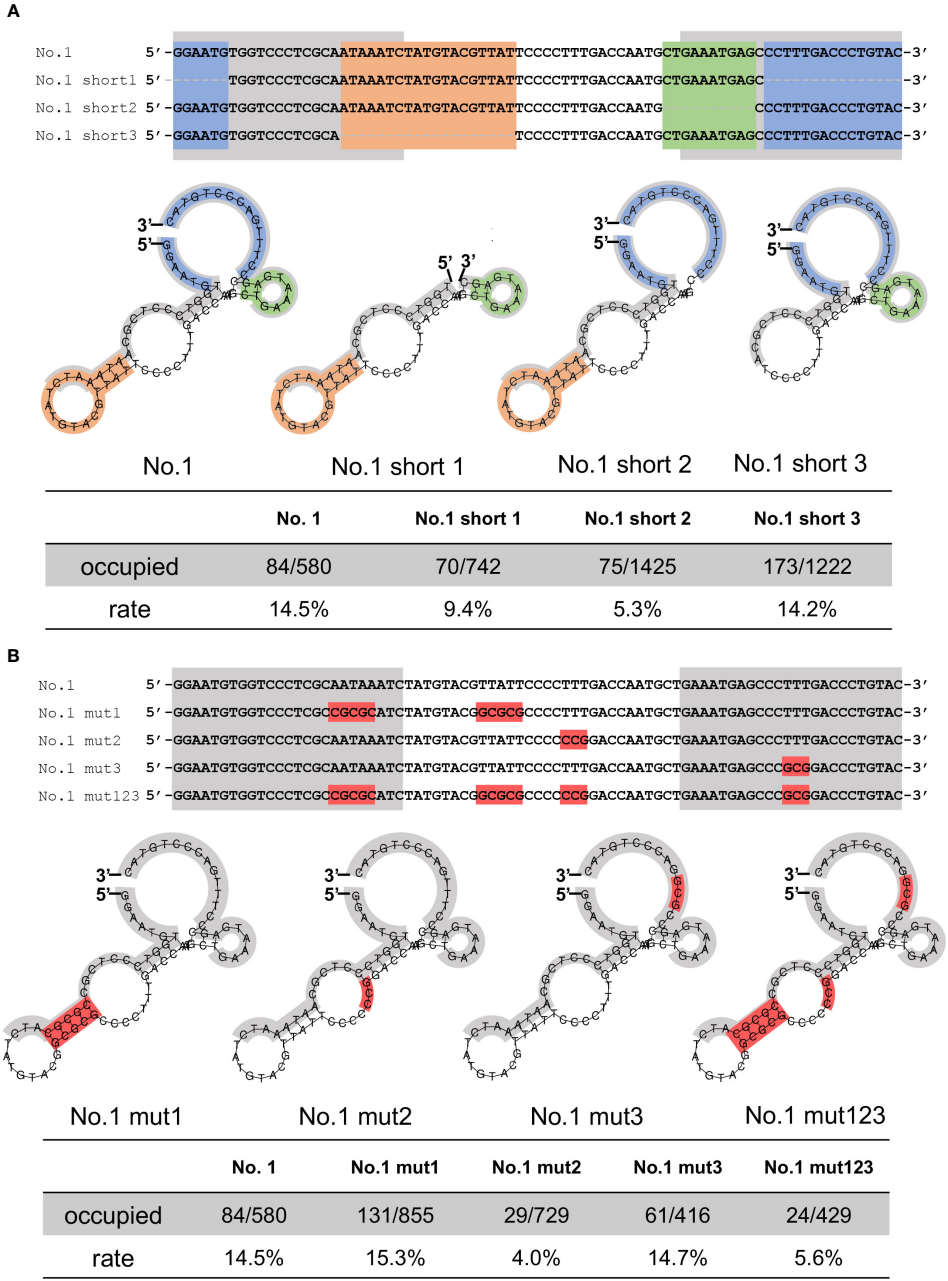
Evaluation of the affinity between truncated or mutated aptamers and NCYM. **(A)** Truncated No.1 aptamers for identification of NCYM binding site. The 5’-3’-end, the central stem loop, the 3’ end stem loop, and the primer region are colored in blue, orange, green, and gray, respectively (upper and middle). Evaluation of the affinity between the truncated aptamers and NCYM (bottom) **(B)** Mutated No.1 aptamers for identification of the NCYM binding site. Mutated sequences are colored in red (upper and middle). Evaluation of the affinity between the mutated aptamers and NCYM (bottom).

Because of the relatively higher stability of the secondary structures of the No.1 aptamer (**Figure S2**), we focused on aptamer No.1 and further analyzed the regions required for NCYM binding. As shown in **Figure 3A**, the AFM results revealed a decrease in the number of bonds in short 1 (9.4%, 70/742 frames) and short 2 (5.3%, 75/1425 frames), whereas there was no change in the number of bonds in short 3 (14.2%, 173/1222 frames). Because aptamers No. 2 and No. 3 exhibited GC-rich sequences (**Figure 1B**), we introduced mutations in the No. 1 aptamer with increasing GC content without affecting the secondary structure, and we found that the mutations at TTT in the central loop showed a significant decrease in the affinity of aptamer No.1 to NCYM (**Figure 3B**). These results suggest that the 5’-3’-end, the 3’ end stem loop, and TTT in the central loop of the No.1 aptamer are required for binding to NCYM.

### 3.3 The effect of DNA aptamer No. 1 on the interaction between NCYM and GSK3β

**Figure 4:**
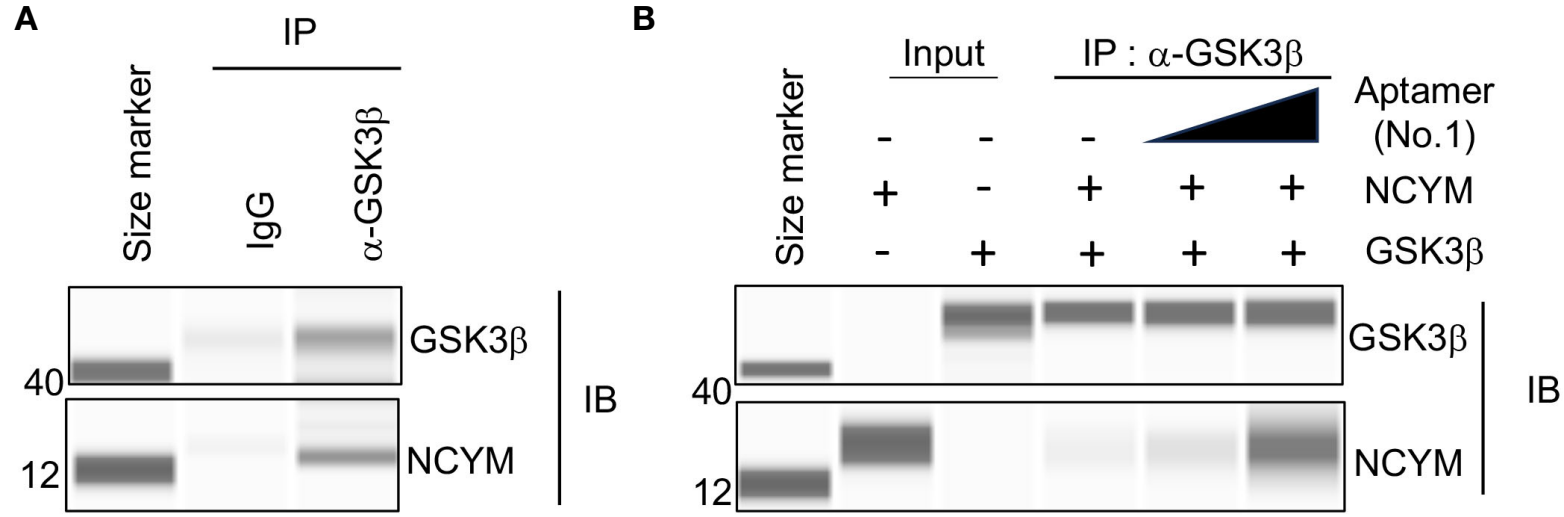
Aptamer No.1 increased interaction between NCYM and GSK3β. **(A)** Co-immunoprecipitation of NCYM with GSK3β detected by Abby analysis. **(B)** Aptamer No.1 increased the amount of NCYM co-immunoprecipitated with GSK3β in a dose-dependent manner (0, 1, and 5 eq.).

To clarify the effect of DNA aptamer No.1 on NCYM function, we examined NCYM binding to GSK3β with or without the aptamer. As reported (1), purified NCYM was co-immunoprecipitated with GSK3β (**Figure 4A**). Addition of aptamer No.1 enhanced the interaction between NCYM and GSK3β in dose-dependent manner (**Figure 4B**). This result led us to analyze the NCYM-aptamer No.1 complex because the aptamer may help NCYM to adopt a conformation that facilitates binding to GSK3β.

### 3.4 SAXS results

#### 3.4.1 Guinier analysis and Kratky plot

**Figure 5:**
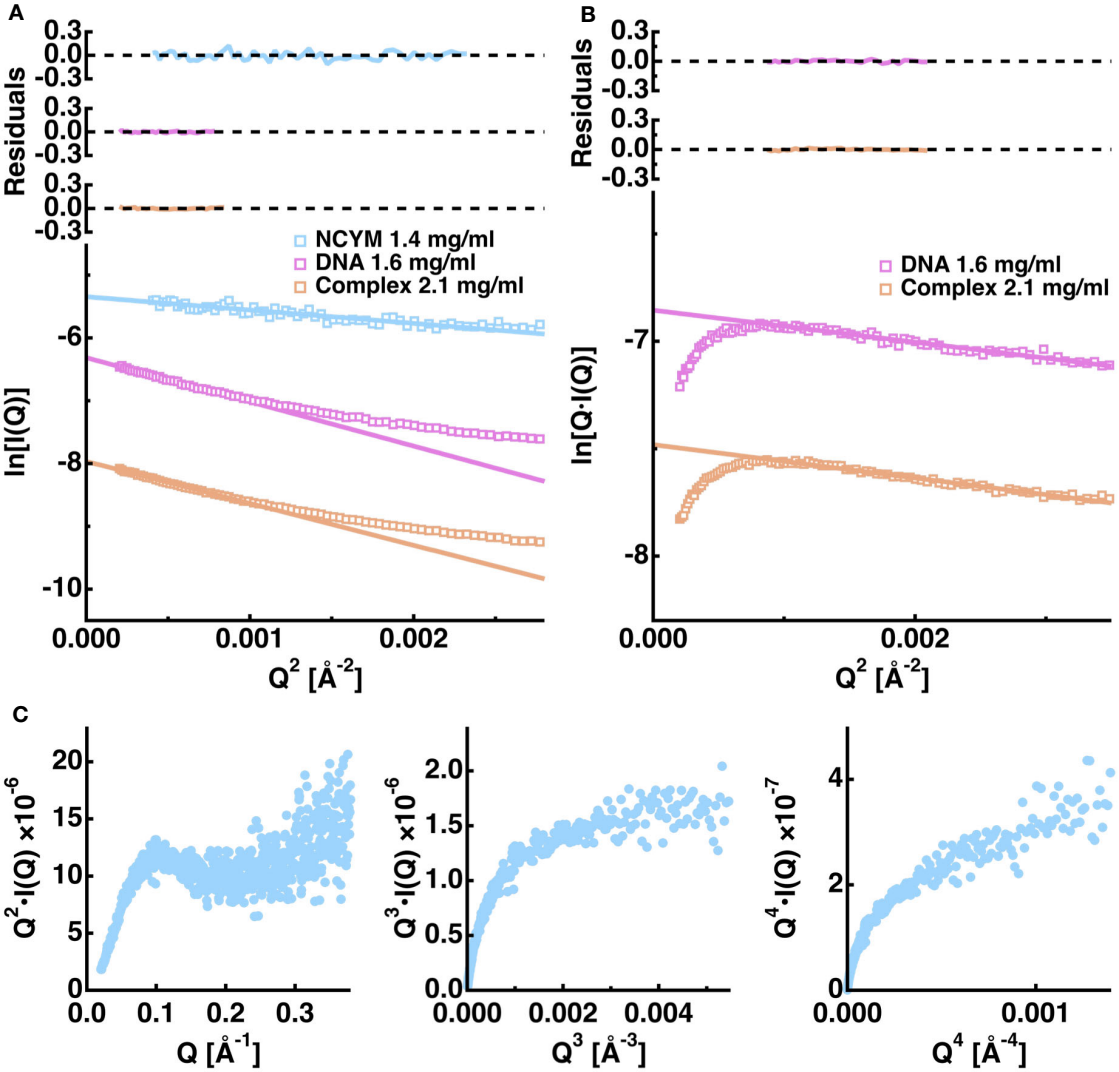
Summary of the analysis of the SAXS scattering curves of the NCYM-DNA system. **(A)** Guinier analysis. The logarithm of the scattering intensity is shown as a function of Q^2^ for NCYM (cyan), the DNA aptamer No. 1 (magenta), and the NCYM-DNA complex (orange). Upper panels denote the corresponding residuals between the measured and the fitted values. **(B)** Cross-sectional Guinier analysis. Instead of ln[I(Q)] of the Guinier analysis, ln[Q･I(Q)] is plotted as a function of Q^2^, from which the cross-sectional radius of gyration is evaluated. **(C)** The left, middle, and right panels show the Kratky plot, Q^3^･I(Q) vs Q^3^ plot, and the Porod-Debye plot, respectively, of NCYM.

To elucidate their structural properties, we employed SAXS analysis to reveal the approximate structures of the complexes in solution. **Figure 5A** shows the results of the Guinier analysis. The radii of gyration (R_g_) were estimated to be 25.2 ± 0.7 Å, 46.0 ± 0.3 Å, and 44.8 ± 0.2 Å, for NCYM, the DNA aptamer No. 1, and the NCYM-DNA complex, respectively. The molecular weights of NCYM and the DNA aptamer were estimated to be 7.2, and 54.5 kDa, respectively, indicating that NCYM molecules exist as a monomer in solution whereas DNA aptamers are dimers because the molecular weights of NCYM and the DNA aptamer were 12 kDa, 24.2 kDa, respectively. Although the molecular weight of the complex was estimated to be 46.8 kDa, because the true concentration of the complexes in the sample is not known due to the existence of a small amount of excess NCYM molecules, this value should be interpreted with caution. Scattering curves other than NCYM were found to follow the cross-sectional Guinier approximation, suggesting that the DNA aptamer and the complex adopt a rod-like shape. **Figure 5B** shows the results of the cross-sectional Guinier analysis, from which the cross-sectional radii of gyration (R_c_) were estimated to be 12.2 ± 0.4 Å and 12.5 ± 0.2 Å for the DNA aptamer and the complex, respectively. This suggests that the overall size of the cross-section is similar between the DNA aptamer and the complex. Both the Guinier analysis and the cross-sectional Guinier analysis can be applied to the current samples because of their relatively short entire length, whereas in many cases, fibrillar proteins are quite long (even on the order of μm) and only the cross-sectional Guinier analysis can be applied (40, 41).

Several plots were used to investigate the structural properties of NCYM, as shown in **Figure 5C**. The Kratky plot (Q^2^･I(Q) vs Q) (42) shows that the Q^2^･I(Q) value reaches a peak at around Q = 0.1 [Å^-1^] and decreases slightly, followed by the increase, suggesting that NCYM is not a completely unfolded protein, but a partially folded protein. This interpretation is further supported by the Q3 plot (Q^3^･I(Q) vs Q^3^), where the Q^3^･I(Q) value reaches a plateau, which is a hallmark of partially folded proteins (43). In contrast, no plateau was observed in the Porod-Debye plot (43), suggesting that NCYM is not a well-folded protein. These results show that NCYM adopts a highly flexible conformation, though it is not a completely unfolded protein, but a partially folded protein. This is in agreement with a previous study showing that NCYM molecules contain defined secondary structures in solution (8).

#### 3.4.2 Solution structure of NCYM, the DNA aptamer, and the NCYM-DNA complex

**Figure 6:**
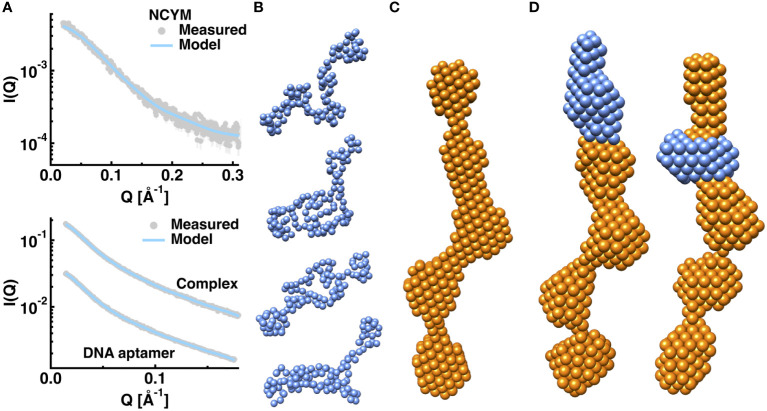
*Ab initio* structural models of NCYM, the DNA aptamer No. 1, and the NCYM-DNA complex. Comparison of the scattering curves between the experiments and the models of NCYM is shown in the upper panel of **(A)** The lower panel of **(A)** shows the comparisons for the DNA aptamer and the NCYM-DNA complex. Experimental values are shown in grey filled circles and the simulated values from the models are shown in cyan solid lines. Error bars are within symbols if not shown. Scattering curves are vertically shifted for clarity. **(B)** Gallery of the NCYM models (arbitrarily chosen 4 models) obtained from GASBOR. Each sphere represents one amino acid residue. **(C)** A representative dummy atom model of the DNA aptamer obtained from DAMMIF and DAMAVER (“damfilt.pdb” is shown). **(D)** Dummy atom models of the NCYM-DNA complex obtained from MONSA. The moieties corresponding to NCYM and the DNA aptamer are shown in marine blue and in orange, respectively.

The results of the structural modeling of NCYM, the DNA aptamer No. 1, and the NCYM-DNA complex are summarized in **Figure 6**. **Figure 6A** compares the experimental SAXS curves with those calculated using the best-fit model obtained for each of the three samples. The χ^2^ values, which are averaged over 10 models obtained, were 1.17, 1.37, and 1.85, for NCYM, the DNA aptamer, and the complex, respectively. As shown in **Figure 6A**, the obtained models provide excellent fits to the measured SAXS curves.

As shown in **Figure 6B**, NCYM was found to have a slightly extended structure in which the bulky and flexible parts are mixed. It is not a completely unfolded protein such as a random coil, as expected from the panels in **Figure 5C** and our previous study on the secondary structure of NCYM (8). Although the assignment of the secondary structure to the three-dimensional structures of the current NCYM models is not possible at this stage, future structural modeling using SAXS curves with higher Q values would be useful for this purpose (44). The volume of the NCYM models was ~17000 Å^3^. Regarding the DNA aptamer, a structure with a volume of ~68000 Å^3^, in which four to five bulky nodes were connected, was found to reproduce the measured SAXS curve, as shown in **Figure 6C**. Because the aptamers exist as dimers, as evidenced by the molecular weight estimation as described above, a monomeric DNA aptamer corresponds to two or three nodes in this model. The structural features of the DNA model with some nodes here are consistent with those predicted using RNAfold (**Figure 1B**). Two representative models of the NCYM-DNA complex are shown in **Figure 6D**. In both cases, the volumes of the NCYM molecule and of the DNA aptamer in the complex were ~19000 Å^3^ and ~63000 Å^3^, which are roughly the same as those obtained when these molecules are in isolation. The volume of the complex (~82000 Å^3^) was essentially the same as the sum of the volumes of NCYM and DNA in isolation (~85000 Å^3^) within 4% accuracy. It thus follows that the NCYM-DNA complex consists of one NCYM molecule and two DNA aptamers (a dimer). NCYM tends to bind either to or close to a tip of a dimeric DNA aptamer. The slight differences (~10%) in the volumes of each component between in isolation and in the complex may imply that some intramolecular structural changes occur upon binding. A more detailed structure of the complex can be obtained by small-angle neutron scattering (SANS) combined with contrast matching or variation (45), which will be performed in the future. All the 10 models obtained for NCYM, the DNA aptamer, and the NCYM-DNA complex are shown in **Figures S4**–**S6**, respectively.

Attempts were made to identify other possible conformations of the complex by changing the volume ratio of each phase in the complex and/or assuming a symmetry in the structure, which are provided as an input file to MONSA. Whereas several models which fit the SAXS curve of the complex quite well in terms of the χ^2^ values were obtained, in all cases, deviation of the volumes of NCYM and the DNA aptamer in the complex with regards to those in the unbound state was much larger (15–20%) than the models presented in **Figure 6**. Considering an independent line of evidence that the volume change of proteins between the folded and the unfolded states is less than 0.5% (46), the volume change of each component in the complex should be the smallest. Since in the models in **Figure 6**, the volume of each component in the complex is close to that in the unbound state, and truncation of one edge of a DNA aptamer breaks down the interaction with NCYM as observed by AFM, the models presented here appear to be reasonable.

## 4 Discussion

In this study, we established a new observation system using aptamers for the single-molecule observation of proteins using DNA origami. To date, protein studies using DNA origami have mainly involved single-molecule observations of proteins that bind directly to DNA or using systems based on ligand or avidin-biotin binding (21–29, 47–52). However, DNA origami research using aptamers has mainly focused on functionalizing the DNA origami using already established aptamers (53–56). Therefore, a system that uses DNA origami and aptamers to elucidate the structure of proteins, as in this study, is a new approach that has never been used. In this study, we have also succeeded in roughly identifying the binding site of NCYM to the aptamer obtained using the SELEX method. The method using aptamers can easily fix proteins onto the DNA origami. Therefore, it is expected to be applied to the observation of the interaction between a fixed protein and its target, and to the functional evaluation of proteins for which single molecule observation has not been performed.

**Figure 7:**
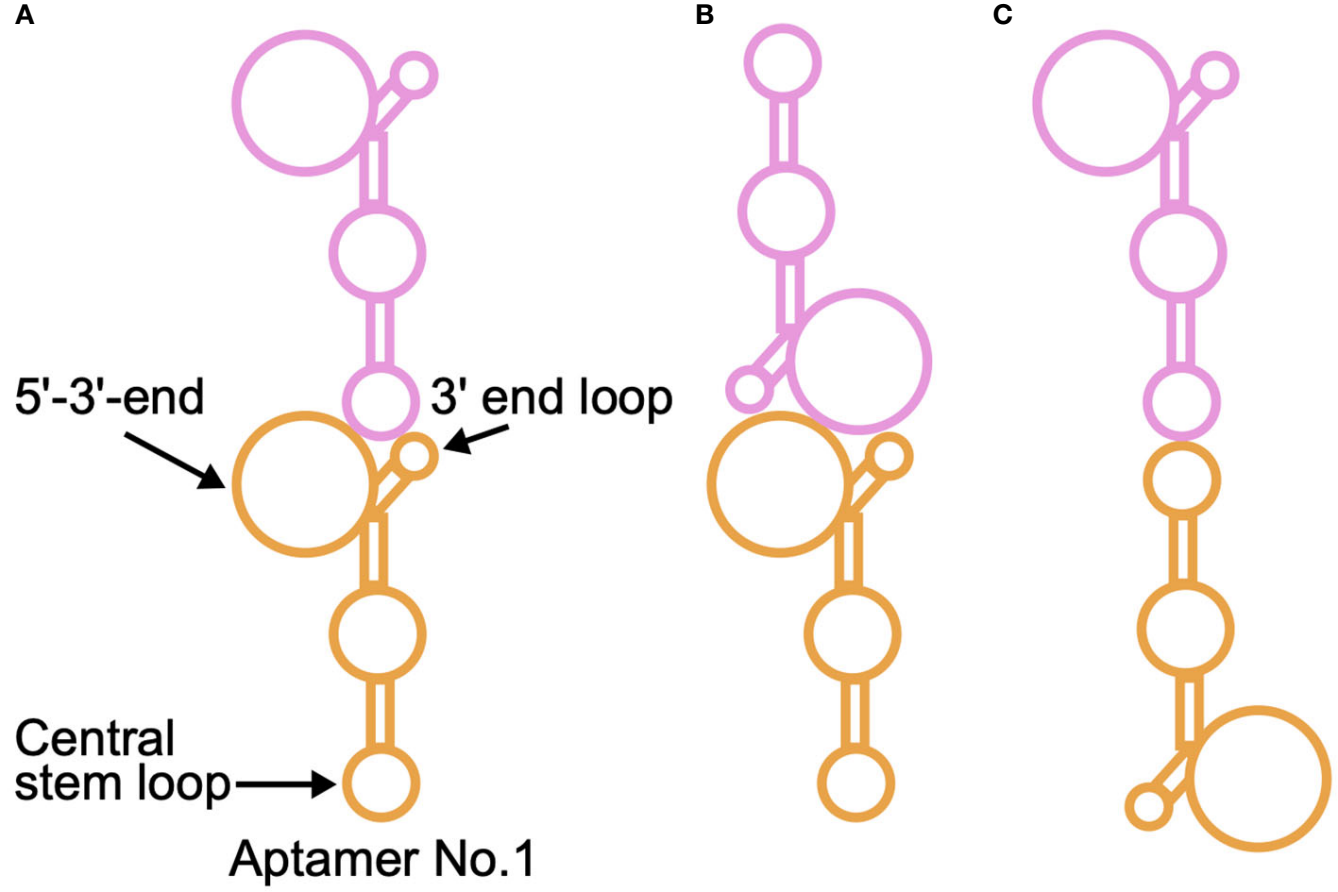
Schematic illustration of possible ways by which the DNA aptamer No. 1 forms a dimer. An aptamer is shown in either orange or magenta. The corresponding predicted structure of the aptamer is shown in **Figure 1B** (left). There are three types of arrangements for two DNA aptamers to take a form similar to that shown in **Figure 6C**: **(A)** The interface between the two aptamers is formed by the central stem loop of one aptamer and the 5’-3’-end of the other aptamer. **(B)** Two 5’-3’-ends form the interface of the dimer. **(C)** The interface of the dimer is formed by two central stem loops. For a more detailed discussion, please see the main text.

In addition to the AFM observation, further structural characterization of NCYM and the DNA aptamer No. 1 that facilitates NCYM binding to GSK3β was conducted using SAXS. Regarding the structure of the DNA aptamer, it was found that it exists as a dimer. This finding is supported by both the molecular weight estimation from the forward scattering intensity and the fitting of the corresponding SAXS curve. Although it is not possible to unambiguously determine the manner in which the two DNA aptamers form dimers, this can be inferred from the current findings. There are three types of arrangements for the two DNA aptamers to form a structure similar to that shown in **Figure 6C** (**Figures 7A–C**).

As shown in **Figure 7**, there are three types of arrangements for two DNA aptamers to form a similar form to that shown in **Figure 6C**. In the case where the interface between the two aptamers is formed by the central stem loop of one aptamer and the 5’-3’-end of the other aptamer (**Figure 7A**), NCYM is able to bind to the 5’-3’-end of the upper aptamer. Whereas there is a possibility that other aptamers bind to either edge of the dimer in the same manner, dimers might be more stable than higher-order aggregates as observed for a protein (57) probably due to entropy-enthalpy compensation. In case where two 5’-3’-ends form the interface of the dimer (**Figure 7B**), NCYM would not be able to bind to the aptamer since these regions are required for NCYM binding as suggested by AFM. If the interface of the dimer is formed by two central stem loops (**Figure 7C**), at least two NCYM molecules would be able to bind to both edges of the dimer, which is inconsistent with the discussion on the SAXS curve of the complex and our modeling results. The second NCYM binding may be unfavorable in terms of entropy. There are thus two possible models on the mode of dimeric formation of the DNA aptamer (**Figures 7A, C**). In addition, the observation that NCYM binds to the dimeric DNA aptamer (**Figure 6**) implies that the affinity between NCYM and an aptamer is lower than that between monomeric aptamers and thus DNA dimers do not dissociate into two monomeric aptamers in the current solution condition.

A concern in the current SAXS analysis is that the deviation of the estimated molecular weight (7.2 kDa) of NCYM from its theoretical value (11.7 kDa) is relatively large. This raises the possibility that the DNA aptamers might exist as trimers or tetramers. The large deviation observed for NCYM is likely to be caused by the small size of NCYM, thus resulting in relatively large experimental errors. However, because the scattering intensity is proportional to the square of the molecular volume, the SAXS curve of the DNA aptamer, which has a larger molecular weight than NCYM, has much lower experimental errors than NCYM (**Figure 6A**). This results in a more reliable molecular weight estimation for the DNA aptamer than for NCYM. Furthermore, the structural modeling of an isolated DNA aptamer presented in this work does not require any information on the molecular property including its molecular weight because it is an “ab initio” approach. From the SAXS-derived models with five bumps (**Figure 6**) and the secondary-structure predicted by RNAfold, which has 2–3 bumps in one aptamer (No. 1 in **Figure 1B**), it is reasonable to conclude that the DNA aptamer forms a dimer in solution.

In the dummy atom models of the complex, the moiety corresponding to NCYM adopted a compact and well-defined shape whereas NCYM showed flexibility and adoped a slightly extended conformation in the unbound state. This implies that highly flexible NCYM molecules fold upon binding to DNA through the well-known “fly-casting mechanism” (58), in which an unfolded region(s) of a protein binds weakly to the binding site at a relatively large distance, followed by folding as the protein approaches the binding site. As aptamer No.1 facilitated the interaction between NCYM and GSK3β, the compact and well-defined shape of NCYM found in the complex with aptamer No. 1 appears to be the functional structure of NCYM. Folding upon binding has been demonstrated for another *de novo* protein AFGP8 (16). A similar mechanism may also apply to interactions between NCYM and GSK-3β, underlying the mechanism of stabilization of these molecules.

One of the most important advantages of AFM observations with a DNA frame is that the DNA frame is guaranteed to be a monomeric aptamer, which is a feature not observed in other binding assays. This is because dsDNA is bound to an DNA aptamer (**Figure 2A**) and thus two dsDNAs should be observed by AFM if the DNA aptamers form dimers, which is not the case. Therefore, it is most likely that monomeric aptamers are capable of binding NCYM and dimer formation of DNA aptamers is not a prerequisite for binding of NCYM. Since the SAXS data alone did not show that NCYM could bind to the monomeric form of the aptamer, AFM, in combination with SAXS, provides important insights into the structure of the aptamer-NCYM complex.

Identification of DNA aptamers that can specifically bind to NCYM and facilitate its binding to GSK3β is useful for elucidation of the structure of NCYM in its active form. Although the aptamer-NCYM complex described in this study has not yet been tested by X-ray crystallography, investigation of the structural properties of the complexes may pave the way for the structural characterization of NCYM molecules at the atomic level. Therefore, the present study suggests that the combination of SELEX, AFM, and SAXS is useful for understanding the structural properties of NCYM and the current findings will serve as a foundation for the future design of novel anti-cancer drugs targeting NCYM as well as for elucidating the stabilization mechanism of other cancer-related proteins by NCYM.

## Data availability statement

The raw data supporting the conclusions of this article will be made available by the authors, without undue reservation.

## Author contributions

YS and TM designed and supervised the study. SY, FK, KN, MH, KH, TT, YH, YS and TM performed the experiments, acquired, and analyzed the data. SY, FK, KN, TT, YS and TM wrote the manuscript. KN, YH, TT, YS and TM acquired funds. All authors contributed to the article, read, and approved the submitted version.
