# Supplementary material for: Structural characterization of human de novo protein NCYM and its complex with a newly identified DNA aptamer using atomic force microscopy and small-angle X-ray scattering
Source: Front Oncol. 2023 Nov 23;13:1213678. doi: 10.3389/fonc.2023.1213678 (PMC10701690; doi:10.3389/fonc.2023.1213678)
Supplement: Supplementary file 1 [file DataSheet_1.docx]

Supplementary Material

Seigi Yamamoto^1^, Fumiaki Kono^2^, Kazuma Nakatani^1,3,4,5^, Miwako Hirose^6^, Katsunori Horii^6^, Yoshitaka Hippo^1,3,7^, Taro Tamada^2,8^, Yusuke Suenaga^1*^, Tatsuhito Matsuo^2*^

^1^Laboratory of Evolutionary Oncology, Chiba Cancer Center Research Institute, Chiba, Japan

^2^Institute for Quantum Life Science, National Institutes for Quantum Science and Technology, Chiba, Japan

^3^Graduate School of Medical and Pharmaceutical Sciences, Chiba University, Chiba, Japan

^4^Innovative Medicine CHIBA Doctoral WISE Program, Chiba University, Chiba, Japan

^5^All Directional Innovation Creator Ph.D. Project, Chiba University, Chiba, Japan

^6^NEC Solution Innovators, Ltd., Tokyo, Japan

^7^Laboratory of Precision Tumor Model Systems, Chiba Cancer Center Research Institute, Chiba, Japan

^8^Graduate School of Science, Chiba University, Chiba, Japan

*** Correspondences:** Yusuke Suenaga: ﻿[ysuenaga@chiba-cc.jp](mailto:ysuenaga@chiba-cc.jp)

Tatsuhito Matsuo: [matsuo.tatsuhito@qst.go.jp](mailto:matsuo.tatsuhito@qst.go.jp)


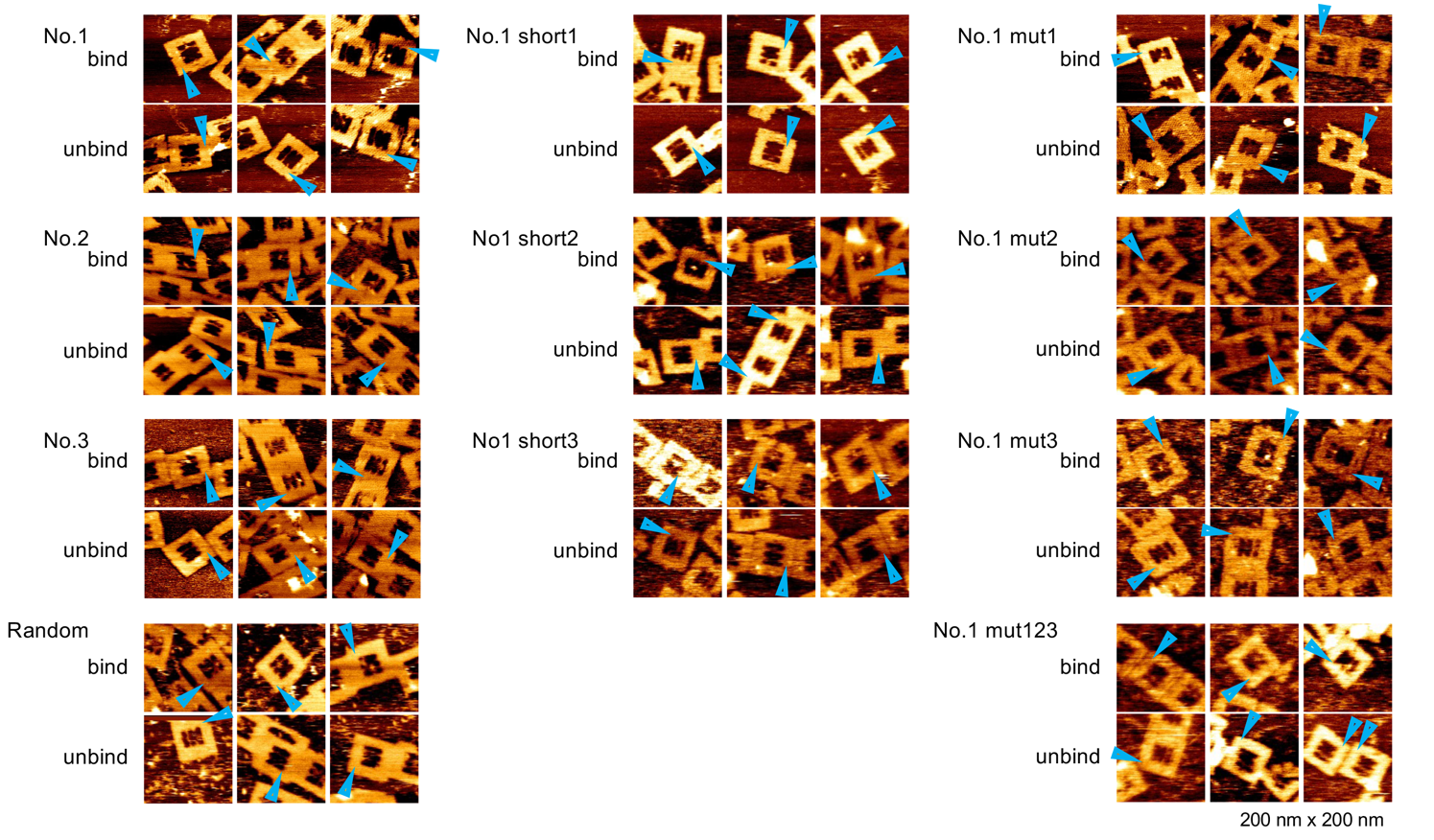


**Figure S1.** Representative AFM images of DNA frame with aptamers. The cyan-color triangles indicate the orientation marker.


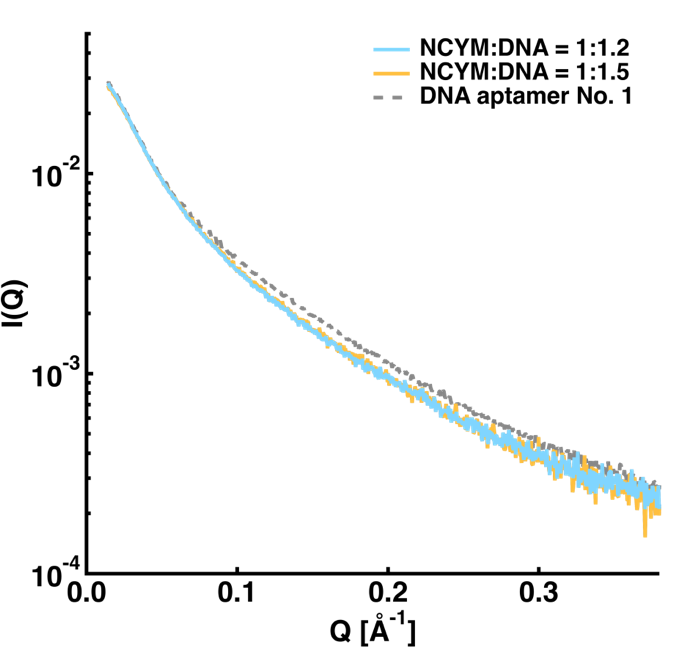


**Figure S2.** Small-angle X-ray scattering curves of the complexes of NCYM and the DNA aptamer No. 1 at the molar ratio of NCYM:DNA = 1:1.2 (cyan), and 1:1.5 (orange). For reference, the scattering curve of the DNA aptamer No. 1 in isolation is shown, which is vertically shifted for comparison. Error bars are not shown for clarity. Two curves of the complex are superimposable quite well across the Q range obtained.


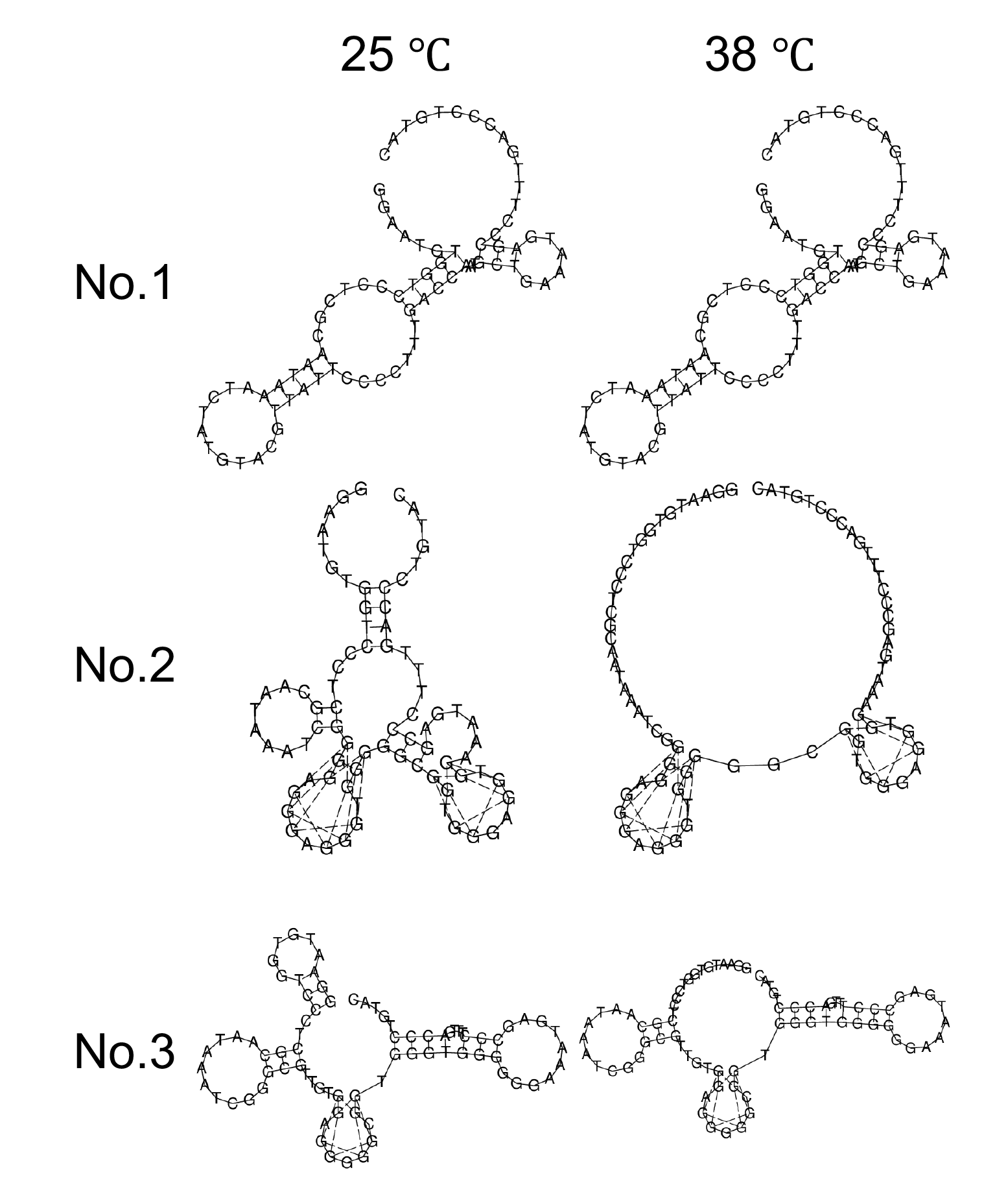


**Figure S3.** Secondary structure prediction model for aptamers at 38°C. Aptamers No. 2 and No. 3 are less thermally stable than No. 1, and different secondary structures are predicted at 25°C and 38°C.


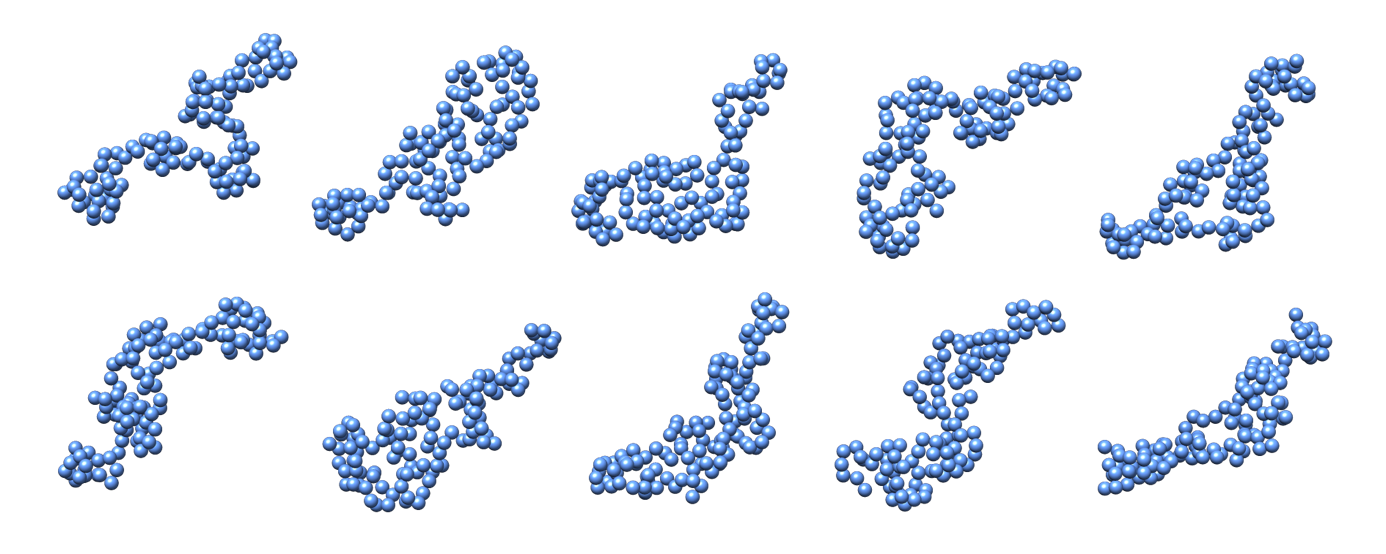


**Figure S4.** Gallery of the dummy-residue models of NCYM in isolation obtained by GASBOR. Results of 10 independent runs are shown. Each sphere represents one amino acid residue.


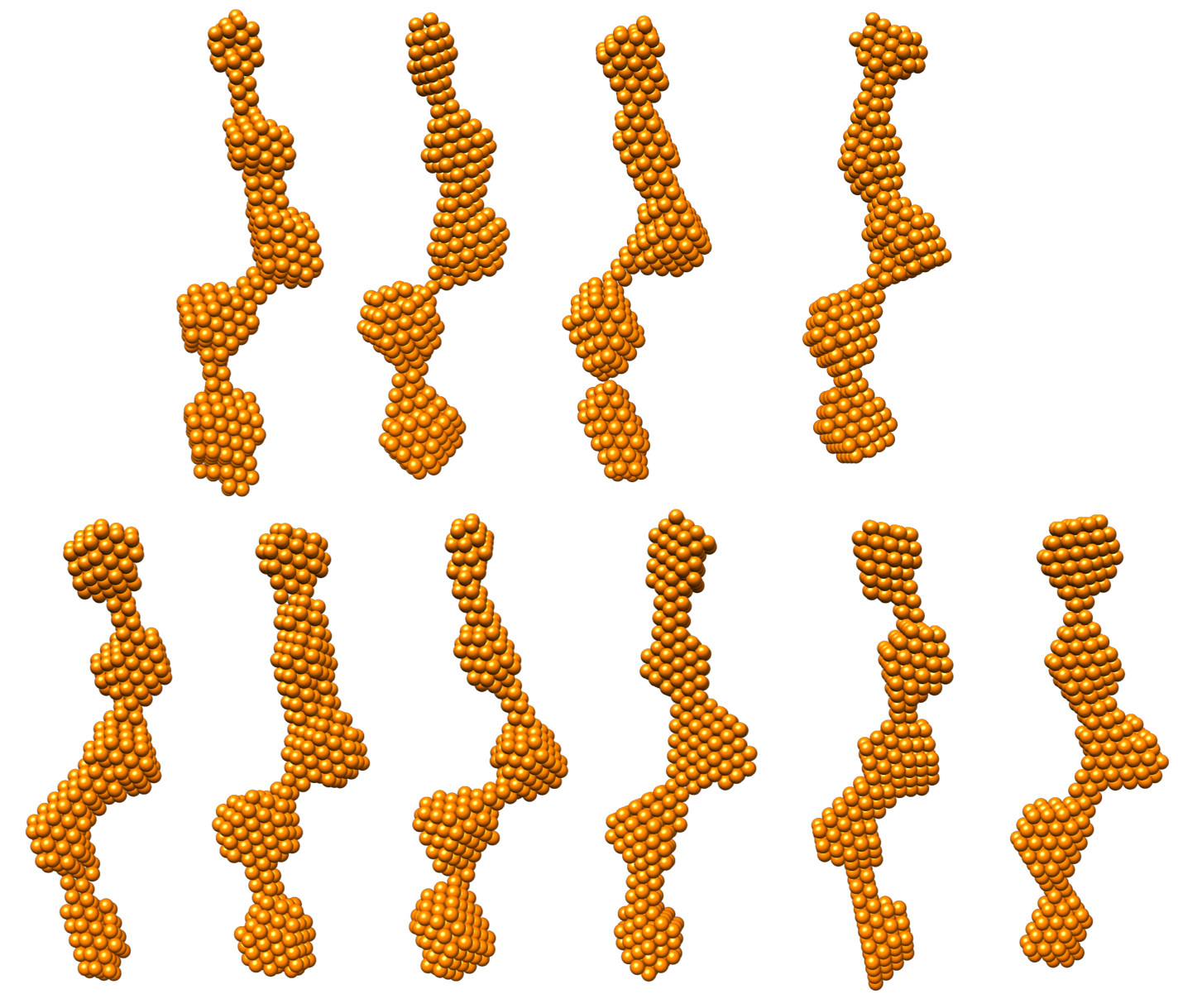


**Figure S5.** Gallery of the dummy-atom models of the DNA aptamer No. 1 in isolation obtained by DAMMIF. Results of 10 independent runs are shown.


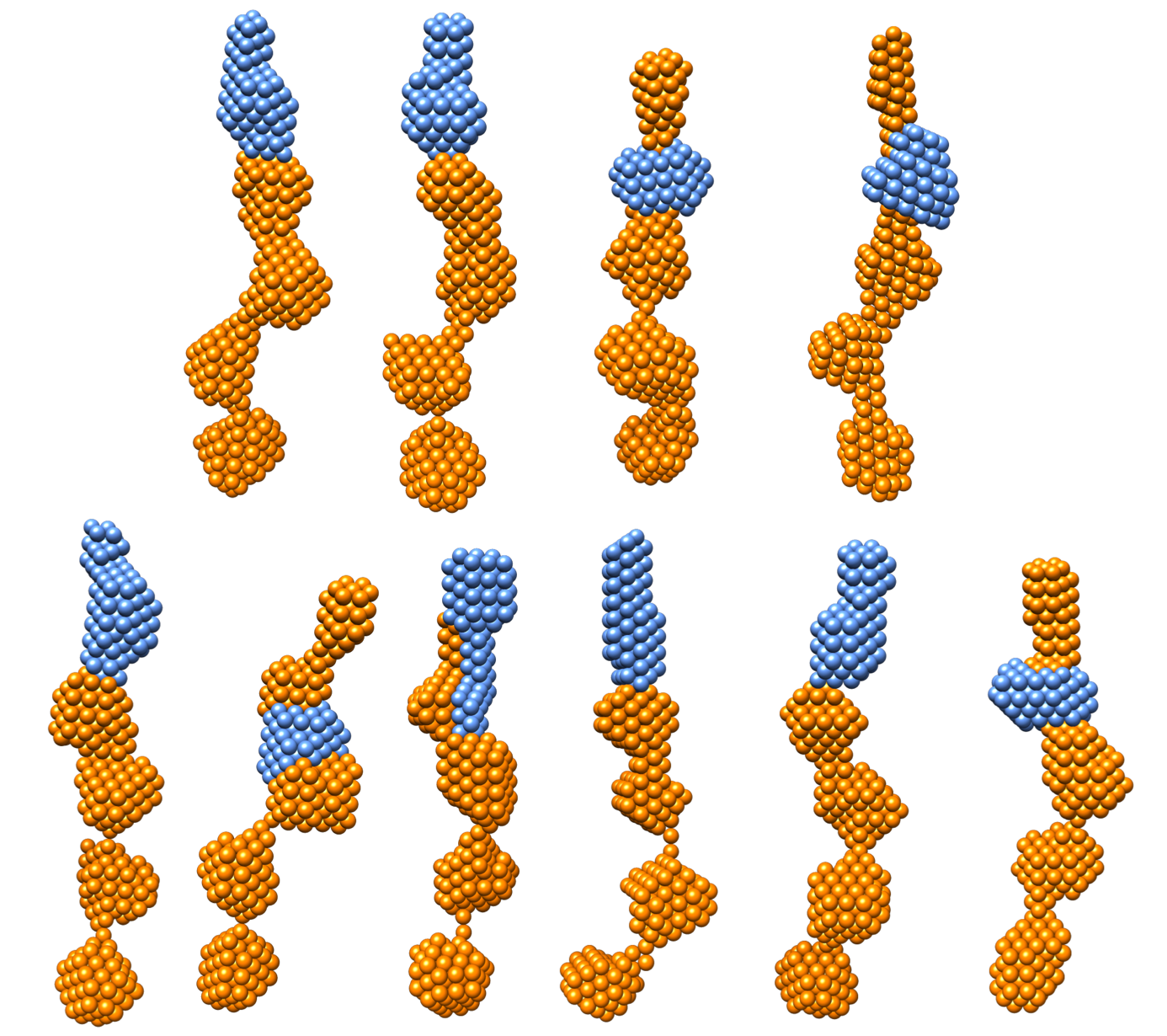


**Figure S6.** Gallery of the dummy-atom models of the NCYM-DNA complex obtained by MONSA. Results of 10 independent runs are shown. The moieties denoted in marine blue and orange correspond to those of NCYM and the DNA aptamer, respectively.

**Table S1.** Number of ssDNA sequences and ACGT contents per rounds after sequence data preparation.

| Selection round | Number of  ssDNA | A (%) | C (%) | G (%) | T (%) |
| --- | --- | --- | --- | --- | --- |
| R3 | 33,797 | 23.2 | 27.2 | 20.3 | 29.3 |
| R4 | 26,904 | 21.8 | 24.4 | 28.0 | 25.8 |
| R5 | 39,574 | 25.2 | 24.4 | 23.4 | 27.0 |
| R6 | 37,372 | 23.5 | 24.0 | 26.7 | 25.7 |
| R7 | 22,905 | 22.0 | 23.1 | 30.4 | 24.5 |
| R8 | 16,590 | 21.0 | 21.8 | 34.5 | 22.7 |

**Table S2.** Kinetic parameters for NCYM and aptamers. Standard error of the means represented as “±SE”.

| Aptamer | *k_a_* (10^3^1/Ms) | *k*_d_ (10^-4^　1/s) | *K_D_* (nM) |
| --- | --- | --- | --- |
| No.1 | 1.14 ± 0.01 | 2.01 ± 0.35 | 176 ± 29.1 |
| No. 2 | 0.86 ± 0.09 | 2.34 ± 0.46 | 298 ± 88.7 |
| No. 3 | 2.31 ± 0.32 | 1.24 ± 0.15 | 53.9 ± 1.06 |
